# α-Synuclein Induces Progressive Changes in Brain Microstructure and Sensory-Evoked Brain Function That Precedes Locomotor Decline

**DOI:** 10.1523/JNEUROSCI.0189-20.2020

**Published:** 2020-08-19

**Authors:** Winston T. Chu, Jesse C. DeSimone, Cara J. Riffe, Han Liu, Paramita Chakrabarty, Benoit I. Giasson, Vinata Vedam-Mai, David E. Vaillancourt

**Affiliations:** ^1^J. Crayton Pruitt Family Department of Biomedical Engineering, University of Florida, Gainesville, Florida 32611; ^2^Department of Applied Physiology and Kinesiology, University of Florida, Gainesville, Florida 32611; ^3^Department of Neuroscience, University of Florida, Gainesville, Florida 32611; ^4^Department of Neurology, University of Florida, Gainesville, Florida 32611

**Keywords:** α-synuclein, diffusion MRI, longitudinal, mouse, resting-state fMRI, sensory-evoked fMRI

## Abstract

*In vivo* functional and structural brain imaging of synucleinopathies in humans have provided a rich new understanding of the affected networks across the cortex and subcortex. Despite this progress, the temporal relationship between α-synuclein (α-syn) pathology and the functional and structural changes occurring in the brain is not well understood. Here, we examine the temporal relationship between locomotor ability, brain microstructure, functional brain activity, and α-syn pathology by longitudinally conducting rotarod, diffusion magnetic resonance imaging (MRI), resting-state functional MRI (fMRI), and sensory-evoked fMRI on 20 mice injected with α-syn fibrils and 20 PBS-injected mice at three timepoints (10 males and 10 females per group). Intramuscular injection of α-syn fibrils in the hindlimb of M83^+/−^ mice leads to progressive α-syn pathology along the spinal cord, brainstem, and midbrain by 16 weeks post-injection. Our results suggest that peripheral injection of α-syn has acute systemic effects on the central nervous system such that structural and resting-state functional activity changes occur in the brain by four weeks post-injection, well before α-syn pathology reaches the brain. At 12 weeks post-injection, a separate and distinct pattern of structural and sensory-evoked functional brain activity changes was observed that are co-localized with previously reported regions of α-syn pathology and immune activation. Microstructural changes in the pons at 12 weeks post-injection were found to predict survival time and preceded measurable locomotor deficits. This study provides preliminary evidence for diffusion and fMRI markers linked to the progression of synuclein pathology and has translational importance for understanding synucleinopathies in humans.

**SIGNIFICANCE STATEMENT** α-Synuclein (α-syn) pathology plays a critical role in neurodegenerative diseases such as Parkinson's disease, dementia with Lewy bodies, and multiple system atrophy. The longitudinal effects of α-syn pathology on locomotion, brain microstructure, and functional brain activity are not well understood. Using high field imaging, we show preliminary evidence that peripheral injection of α-syn fibrils induces unique patterns of functional and structural changes that occur at different temporal stages of α-syn pathology progression. Our results challenge existing assumptions that α-syn pathology must precede changes in brain structure and function. Additionally, we show preliminary evidence that diffusion and functional magnetic resonance imaging (fMRI) are capable of resolving such changes and thus should be explored further as markers of disease progression.

## Abstract (precis)

*In vivo* functional and structural brain imaging of synucleinopathies in humans have provided a rich new understanding of the affected networks across the cortex and subcortex. Despite this progress, the temporal relationship between α-synuclein (α-syn) pathology and the functional and structural changes occurring in the brain is not well understood.

## Introduction

Intracellular aggregation of α-synuclein (α-syn) has been associated with a group of diseases known as synucleinopathies, which include Parkinson's disease, multiple system atrophy, and Lewy body dementia. Although α-syn aggregates are reliably found in patients and are the target of many therapeutic approaches (Sardi et al., 2018), the temporal and pathophysiological relationships between α-syn pathology, neurodegeneration, and clinical symptoms are not well understood. It has been hypothesized that, in Parkinson's disease, retrograde propagation of α-syn pathology occurs along synaptically coupled pathways resulting in neuron dysfunction and death (Braak et al., 2004). However, evidence has arisen that additional mechanisms may influence the propagation and effects of α-syn pathology (Surmeier et al., 2017). For example, postmortem analyses of Lewy body dementia and Parkinson's disease cohorts have reported neurodegeneration of nigral neurons that precedes α-syn pathology in the nigrostriatal system (Milber et al., 2012; Dijkstra et al., 2014), yet a systematic characterization of the temporal occurrence has yet to occur.

In an effort to elucidate a greater understanding of α-syn aggregation and its effects, an α-syn fibril seeded mouse model was generated that robustly develops α-syn pathology initiated by intramuscular injection of α-syn fibrils in the biceps femoris (Sacino et al., 2014). Human Ala53Thr hemizygous (M83^+/−^) α-syn transgenic (Tg) mice intramuscularly injected with α-syn fibrils develop α-syn pathology throughout the spinal cord and brain leading to bilateral hindlimb paralysis at four months post-injection (Sacino et al., 2014; Sorrentino et al., 2018). Without the intramuscular injection of α-syn fibrils, these mice do not develop symptoms or pathology until 22–28 months of age (Giasson et al., 2002). In this study, we used this seeded model of synucleinopathy to examine the progressive changes in the microstructural degeneration and functional activation of brain circuits in the context of progressive induction of α-syn pathology in the neuroaxis. This is a particularly relevant question since brain microstructure and functional activation are established longitudinal markers of progression in patients with Parkinson's disease (Burciu et al., 2016; Archer et al., 2019).

Magnetic resonance imaging (MRI) is a non-invasive *in vivo* imaging technique that can be applied to characterize the structural and functional properties of the brain. For example, fractional anisotropy is a diffusion MRI (dMRI) metric that has been used to quantify tissue density and white matter integrity (Le Bihan et al., 2001). Blood oxygen level-dependent (BOLD) signal is a function MRI metric that represents populations of neuronal activity in relation to resting and task-activation states (Ogawa et al., 1992; De Luca et al., 2006). While many studies have used MRI to examine structural and functional characteristics of human patients with synucleinopathies (Seppi et al., 2003; Cochrane and Ebmeier, 2013; Lewis et al., 2018), we do not yet understand the longitudinal progression of microstructure and functional activation of the brain that results from α-syn pathology (Helmich et al., 2018).

**Figure 1.**
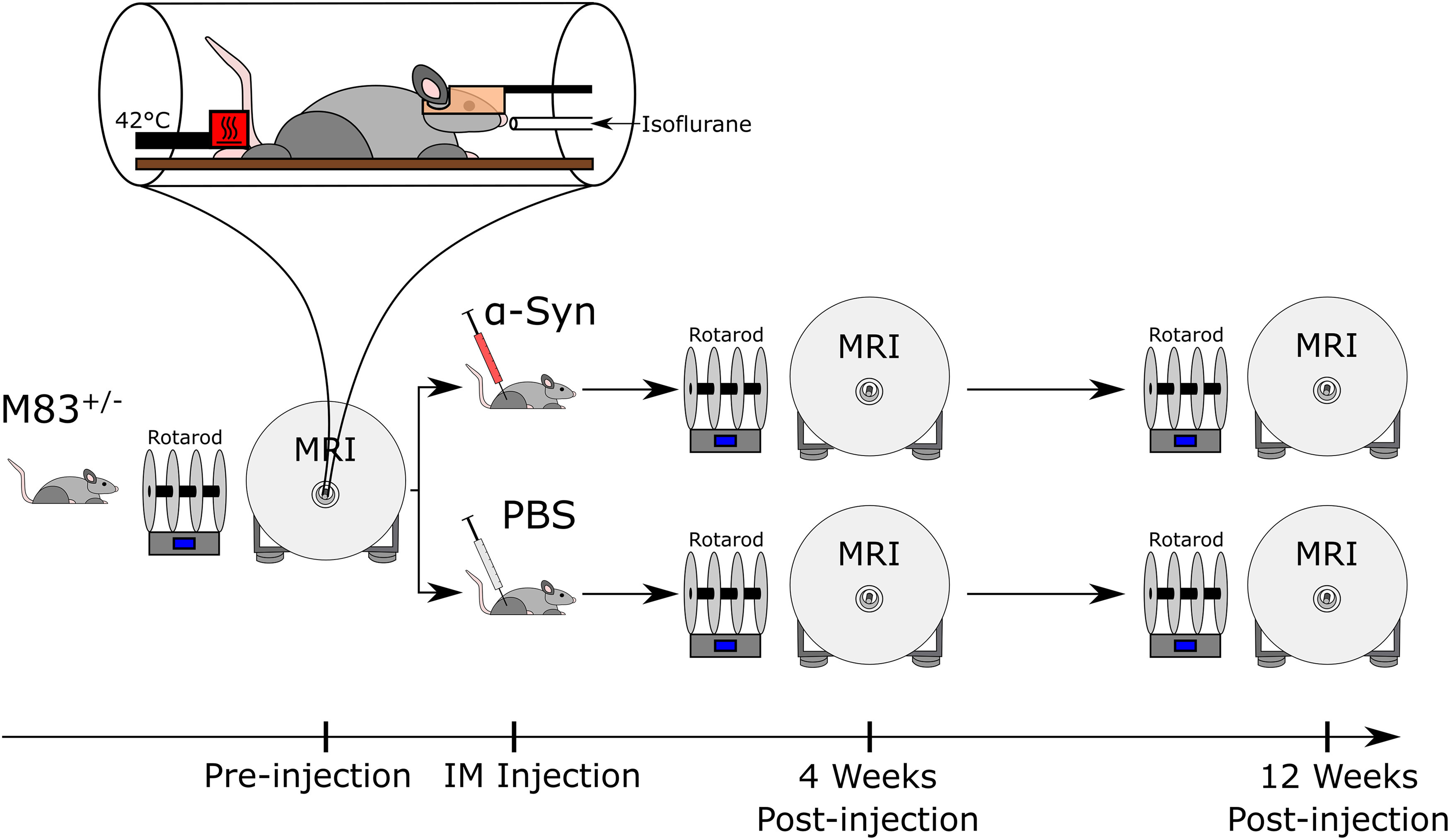
Experimental timeline. M83^+/−^ mice were differentiated into α-syn and control groups via intramuscular injection of either α-syn fibrils or PBS, respectively. *In vivo* dMRI, sensory-evoked fMRI, and resting-state fMRI were collected pre-injection, four weeks post-injection, and 12 weeks post-injection on all mice. A heat stimulation of 42°C was performed on the hind paw in a block paradigm during sensory-evoked fMRI. Rotarod locomotor assessments were performed in the week preceding imaging.

In this study, dMRI, resting-state functional MRI (fMRI), and sensory-evoked fMRI assays were used to longitudinally investigate the progression of microstructure and functional activation changes occurring in an α-syn transmission mouse model. Specifically, all three types of MRI scans were collected in a group of 20 M83^+/−^ mice (10 male/10 female) that received bilateral intramuscular injections of 10 μg of α-syn fibrils and a vehicle control group of 20 M83^+/−^ mice (10 male/10 female) that received bilateral intramuscular injections of phosphate-buffered saline (PBS). Using an 11.1 Tesla horizontal bore MRI, we imaged the mice at three timepoints: pre-injection (baseline), four weeks post-injection, and 12 weeks post-injection (Fig. 1). Rotarod locomotor assessments were also performed on all mice in the 4 d preceding each of the imaging timepoints. Cox proportional hazards regression models were created to determine which metrics and which regions were most predictive of survival time. The findings provide the first insight into how in vivo microstructure and functional brain activity progresses in an α-syn mouse model and establishes a link between α-syn, brain microstructure, and function.

## Materials and Methods

### 

#### Animals

All animals were maintained on a 12/12 h light/dark cycle with food and water *ad libitum*. Animals were acquired and cared for in accordance with the National Institutes of Health *Guide for the Care and Use of Experimental Animals*. All procedures were approved by the University of Florida Institutional Animal Care and Use Committee. At two months of age, human Ala53Thr hemizygous (M83^+/−^) α-syn Tg mice (Giasson et al., 2002) received bilateral intramuscular (biceps femoris) injections of 10 μg of full-length (wild-type) mouse α-syn fibrils or 5 μl of sterile PBS. M83^+/−^ Tg mice were maintained on a C57Bl/C3H background strain. Both groups contained equal numbers of males and females. There were no significant between-group differences in weight at any of the three timepoints. The weight of mice injected with α-syn fibrils ranged from 19.7 to 48.8 g with a mean of 31.1 g, while the weight of PBS-injected mice ranged from 18.9 to 46.9 g with a mean of 28.9 g.

The intramuscular seeding and CNS neuroinvasion α-syn mouse model was developed and first described in Sacino et al. (2014) and has been shown to robustly develop α-syn pathology and hindlimb paralysis within ∼16 weeks of injection of the α-syn fibrils (Ayers et al., 2017; Sorrentino et al., 2018). Importantly, it has also been shown that M83^+/−^ Tg mice do not endogenously develop α-syn pathology before 22 months of age (Giasson et al., 2002). The α-syn pathology mouse model examined in this study has been characterized using histology at pre-injection, one month, and three months post-injection (Sorrentino et al., 2018). Specifically, α-syn pathology, microgliosis, and astrogliosis were measured in the spinal cord, pons, midbrain, thalamus, and cortex. α-Syn pathology, microgliosis, and astrogliosis were not detected to occur in the examined regions by one month post-injection. α-Syn pathology was observed in the spinal cord, pons, midbrain, thalamus, and cortex at three months post-injection. Microgliosis and astrogliosis were detected in the spinal cord and pons at three months post-injection.

The examined α-syn pathology mouse model holds some similarities to sporadic and familial Parkinson's disease. The background strain (Tg M83^+/−^) of the disease and control group contains the A53T missense mutation found in a form of familial Parkinson's disease. After the injection of α-syn fibrils, it has been observed that the pathology spreads anteriorly up the spinal cord to the brain (Sacino et al., 2014; Sorrentino et al., 2018). Although this model is useful for studying the spread and progression of α-syn pathology, it should not be construed as a direct model of Parkinson's disease. For example, Parkinson's disease patients do not experience hindlimb paralysis at any point in the disease progression. Additionally, α-syn pathology does not preferentially aggregate in the substantia nigra in these mice.

#### Experimental design

Two groups of M83^+/−^ Tg mice were examined in this study: mice intramuscularly injected with α-syn preformed fibrils (*n* = 20), or genotype-matched mice intramuscularly injected with PBS (*n* = 20). Mice were randomly assigned to experimental groups and group assignment was stratified across the duration of the study. MRI scans were collected on all mice at three timepoints: pre-injection, four weeks post-injection, and 12 weeks post-injection. Intramuscular injections occurred within 24 h after the pre-injection scans were collected. Four types of MRI scans were collected for every mouse at every time point: dMRI, an anatomic scan, resting-state fMRI, and sensory-evoked fMRI. A rotarod assessment was conducted on all mice at each of the three timepoints in the 4 d preceding the scan to broadly quantify locomotor function. Figure 1 contains a schematic of the timeline of experiments relative to the group-defining injection.

#### MRI equipment

MRI experiments were conducted on an 11.1 Tesla Magnex Scientific horizontal bore magnet (Agilent; BFG-240/120-S6 gradient system with 120-mm inner gradient bore size; maximum gradient strength 1000 mT/m and rise time of 200 μs) interfaced to a Bruker Avance III HD console and controlled by Paravision 6.01 software (Brucker BioSpin) at the McKnight Brain Institute, University of Florida. Imaging was collected using an in-house 2.5 × 3.5 cm quadrature surface transmit/receive coil affixed to the top of the skull and tuned to 470.7 MHz (H^1^ resonance) for B^1^ excitation and signal detection (AMRIS Facility, University of Florida).

#### MRI data acquisition

Mice were anesthetized for the duration of the experiment. Isoflurane anesthesia was delivered using compressed medical-grade air (70% N_2_/30% O_2_) through a Surgivet vaporizer connected to a charcoal trap. Mice were induced at 3% isoflurane for 1–2 min in an enclosed knock-in chamber. Anesthesia is reduced to 2% for animal setup and was maintained between 1.0% and 1.5% for MRI acquisition. Notably, this concentration of isoflurane is within a range that has been shown to preserve functional connectivity patterns in rodent models (Ferron et al., 2009; Liu et al., 2013). Mice were placed in a prone position on a custom 3D-printed plastic mouse bed equipped with a bite bar that serves to immobilize the head and deliver anesthesia during scanning. Respiratory vitals were monitored using a respiration pad and core body temperature was maintained at 36–37°C (Reimann et al., 2016) using a recirculating water heating system (SA Instruments).

#### dMRI scan parameters

dMRI with echoplanar imaging (EPI) distortion correction scans were acquired with the following parameters: repetition time (TR) = 4000 ms; echo time (TE) = 19.17 ms; slices = 17; coronal orientation; thickness =0.7 mm; gap = 0 mm; FOV = 15 × 11 mm; data acquisition matrix = 128 × 96 in-plane; two b0 images; b values = 600 and 2000 s/mm^2^; directions = 52 total (six at b value = 600 s/mm^2^; 46 at b value = 2000 s/mm^2^). Time between gradient pulses: 8 ms; diffusion gradient duration: 3 ms.

#### fMRI scan parameters

fMRI scans were each performed using a two-shot EPI sequence with the following parameters: TR = 2000 ms; TE = 15 ms; repetitions = 180 (resting-fMRI) or 360 (sensory-fMRI); flip angle = 90°; dummy scans = 2; slices = 13; coronal orientation; thickness = 0.9 mm; gap = 0 mm; FOV = 15 × 15 mm; data acquisition matrix = 64 × 64 in-plane. Anatomical images were acquired using a fast-spin echo T2-weighted imaging sequence with the following parameters: TR = 5500 ms; effective echo time (TE_eff_) = 30.1 ms; averages = 7; slices = 13; coronal orientation; thickness = 0.9 mm; gap = 0 mm; time between echoes = 3.8 ms; rare factor = 16; FOV = 15 × 15 mm; data acquisition matrix = 256 × 256 in-plane.

#### Temperature stimulation paradigm

During the sensory-evoked procedure, thermal stimulation was applied to the right plantar hind paw. The heating thermode (Pathway Model ATS; Medoc Ltd.) delivered heat to a 15 × 15 mm area and was positioned such that the tips of the toes aligned with the far edge of the heated surface, maximizing the contact area between the hind paw and the heated surface. The heating thermode was calibrated to produce baseline and thermal stimulation temperatures of 30°C and 42°C, respectively. This stimulation temperature was chosen so as not to elicit pain (Becerra et al., 2011; Bosshard et al., 2015; Reimann et al., 2016). During each 12-min scan, thermal stimulation was applied in a block paradigm (five stimulation blocks total), alternating between 60 s at the stimulation temperature (42°C) and 60 s at the baseline temperature (30°C). Each scan began and ended with a 30-s baseline block. The change in temperature between blocks was achieved within 300 ms via a cooling rate of 40°C/s and a heating rate of 70°C/s.

#### Rotarod

The rotarod task assesses locomotor function and was conducted using a Rota-Rod treadmill Model 7650 (Ugo Basile). The assessment occurred over four consecutive days: 2 d of training followed by 2 d of testing. On each day, the mice performed three trials. Each trial is 5 min long, during which the rotarod accelerated from 4 to 40 revolutions per minute. Mice were placed on the rod and the latency to fall was recorded, with a max score of 300 s. Each trial was followed by a 10-min rest period to reduce the effects of fatigue.

#### dMRI processing

**Figure 2.**
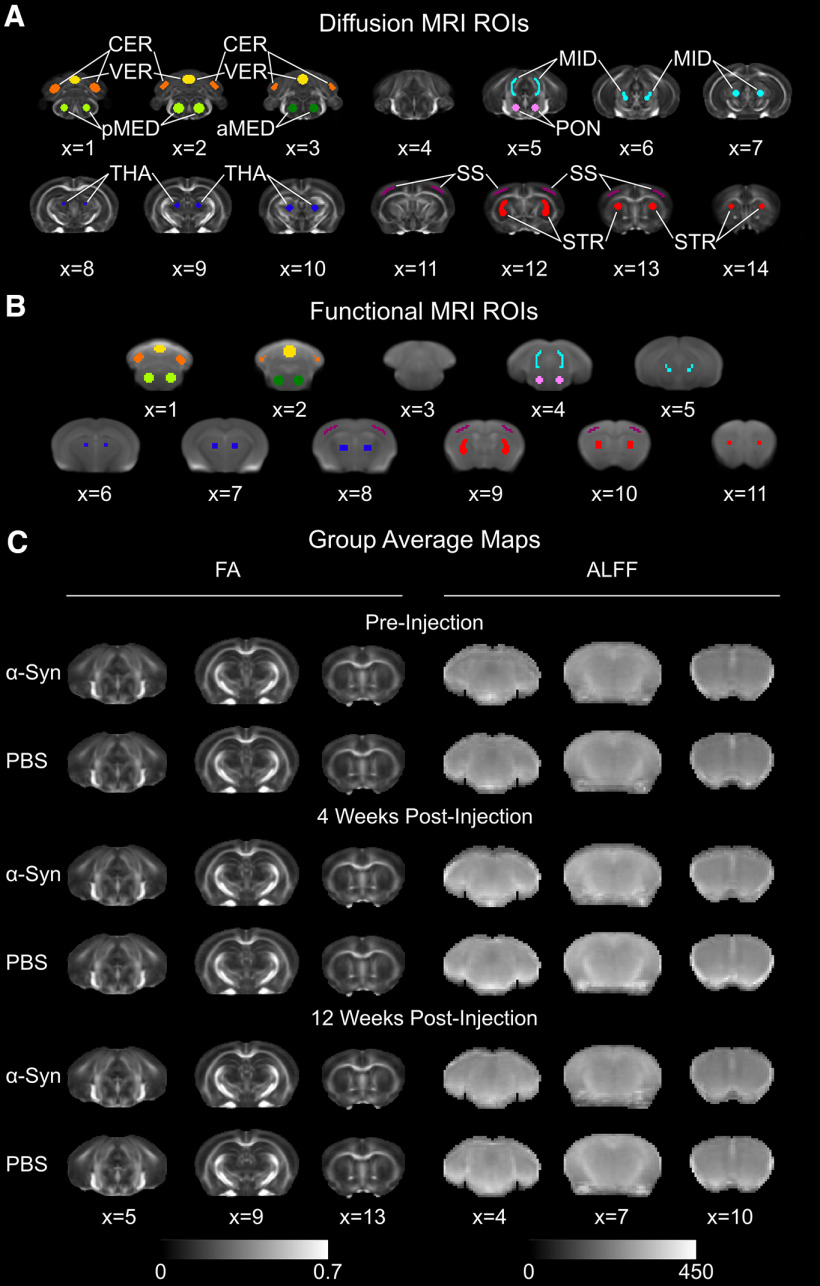
ROIs and group average maps. ***A***, dMRI ROIs overlaid on top of the fractional anisotropy (FA) template image: cerebellum (orange), vermis (yellow), posterior medulla (light green), anterior medulla (dark green), pons (pink), midbrain (turquoise), thalamus (dark blue), somatosensory cortex (purple), striatum (red). ***B***, fMRI ROIs. The underlay is a representative temporal mean resting-state fMRI image averaged across subjects. The color scheme of the ROIs matches that of ***A***. ***C***, Representative slices from the group average FA and ALFF maps for all three time points.

**Figure 3.**
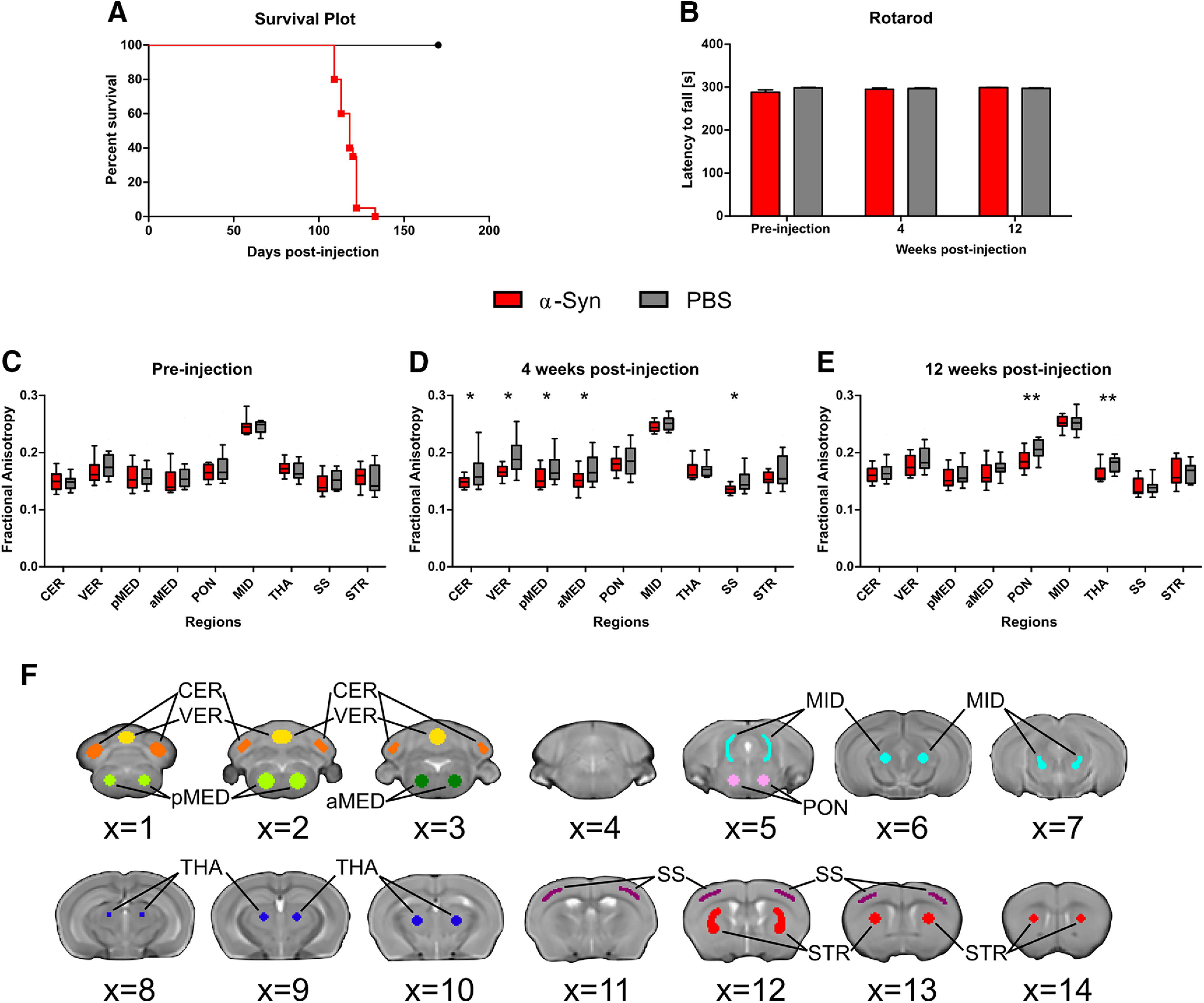
Intramuscular injection of α-syn fibrils induces structural changes in the brain and death. ***A***, Kaplan–Meier survival plot comparing survival times of mice intramuscularly injected with α-syn to PBS-injected control mice. ***B***, Bar plot shows mean latency to fall of the six rotarod test trials at each of the three examined timepoints. Between-group differences in latency to fall were not statistically significant. Error bars designate SEM. ***C–E***, Box plots show the interquartile range for fractional anisotropy measured using diffusion-weighted MRI pre-injection (***C***), four weeks post-injection (***D***), and 12 weeks post-injection (***E***). Fractional anisotropy was calculated in α-syn and PBS control mice in ROIs: cerebellum (CER), vermis (VER), posterior medulla (pMED), anterior medulla (aMED), pons (PON), midbrain (MID), thalamus (THA), somatosensory cortex (SS), striatum (STR). Regions with significant differences between groups after FDR correction are designated with asterisk(s) (*p_fdr_ < 0.05; **p_fdr_ < 0.01). ***F***, Hand-drawn ROIs used in dMRI analyses. Voxels in the center of each region were chosen to make results robust to minor registration errors, common to scans with severely anisotropic voxel sizes. Regions were drawn on and are displayed overlaid over the B0 template: cerebellum (orange), vermis (yellow), posterior medulla (light green), anterior medulla (dark green), pons (pink), midbrain (turquoise), thalamus (dark blue), somatosensory cortex (purple), striatum (red).

dMRI images were processed using the FMRIB Software Library (FSL) (Smith et al., 2004) and custom UNIX shell scripts. The dMRI processing pipeline was completely automated and could, therefore, be applied equally and without bias to all scans. All scans were corrected for distortions because of eddy currents and head motion using affine transforms. The gradient directions were rotated in response to these corrections (Leemans and Jones, 2009), and non-brain tissue was removed. Diffusion tensor imaging maps (i.e., fractional anisotropy; mean diffusivity) were then calculated (Basser et al., 1994). To compare regional differences between mice with varying brain size and shape, the fractional anisotropy and mean diffusivity maps were registered to a template image. All registration was performed using the advanced normalization tools (ANTs) toolkit (Avants et al., 2008). The diffusion scans had a high in-plane resolution (117 × 115 μm) with a relatively large slice thickness (700 μm). Thus, to prevent interslice warping, each slice was independently and nonlinearly registered to the matching template image slice. Group average FA maps in template space are shown in Figure 2*C* and illustrate the image quality and registration quality. The fractional anisotropy images were registered to the fractional anisotropy template and the transformations were applied to the mean diffusivity images. To create the template image, 160 fractional anisotropy images were iteratively averaged, registered to the average, then averaged again using the antsMultivariateTemplateConstruction.sh script in the ANTs toolkit. This custom template allowed us to register our images to a common coordinate space while minimizing the magnitude of the transformations calculated, thus maximizing the registration accuracy. A single set of regions of interest (ROIs) were hand-drawn on the B0 template image and are shown in Figure 2*A* overlaid on the FA template and in Figure 3*F* overlaid on the B0 template. ROIs were drawn in the center of each region to make the results robust to registration errors, common in anisotropic scans. Regions were chosen based on size and significance in α-syn pathology (Sacino et al., 2014; Sorrentino et al., 2018). These regions include the cerebellum, vermis, medulla oblongata, pons, midbrain, thalamus, cortex, and striatum. ROIs were applied to the fractional anisotropy and mean diffusivity maps in template space to calculate the mean fractional anisotropy and mean diffusivity values within each ROI.

#### fMRI preprocessing

Sensory-evoked and resting-state fMRI images were processed using the AFNI software package (Analysis of Functional NeuroImages; National Institutes of Health), and custom UNIX shell scripts. Except for manual brain extraction of the anatomic images, the fMRI processing pipeline was completely automated and could, therefore, be applied equally and without bias to all scans. The first five volumes were removed from each functional scan to allow for equilibration of the T_1_ signal and duplicate scans were concatenated. Outliers in each voxel's time series were identified using 3dToutcount and volumes with >5% of voxels calculated to be outliers were flagged for exclusion during the regression step. The functional images were then despiked, slice acquisition-dependent slice-time corrected, motion-corrected, spatially smoothed (0.3-mm full width half maximum Gaussian blur) and scaled to have a range of (0,200) and a mean of 100. The transformation matrix calculated from the motion-correction step was used to compute de-meaned and derivatives of motion parameters for use as regressors. Volumes in which the Euclidian norm of the derivative values exceeded 0.5 were flagged for exclusion during the regression analysis. Whole-brain masks were created by registering each mouse's anatomic image to the mean of the functional scan and applying the transformations to a manually drawn anatomical mask. The resulting image was binarized and applied to the functional scan to remove the signal from non-brain tissue.

**Figure 4.**
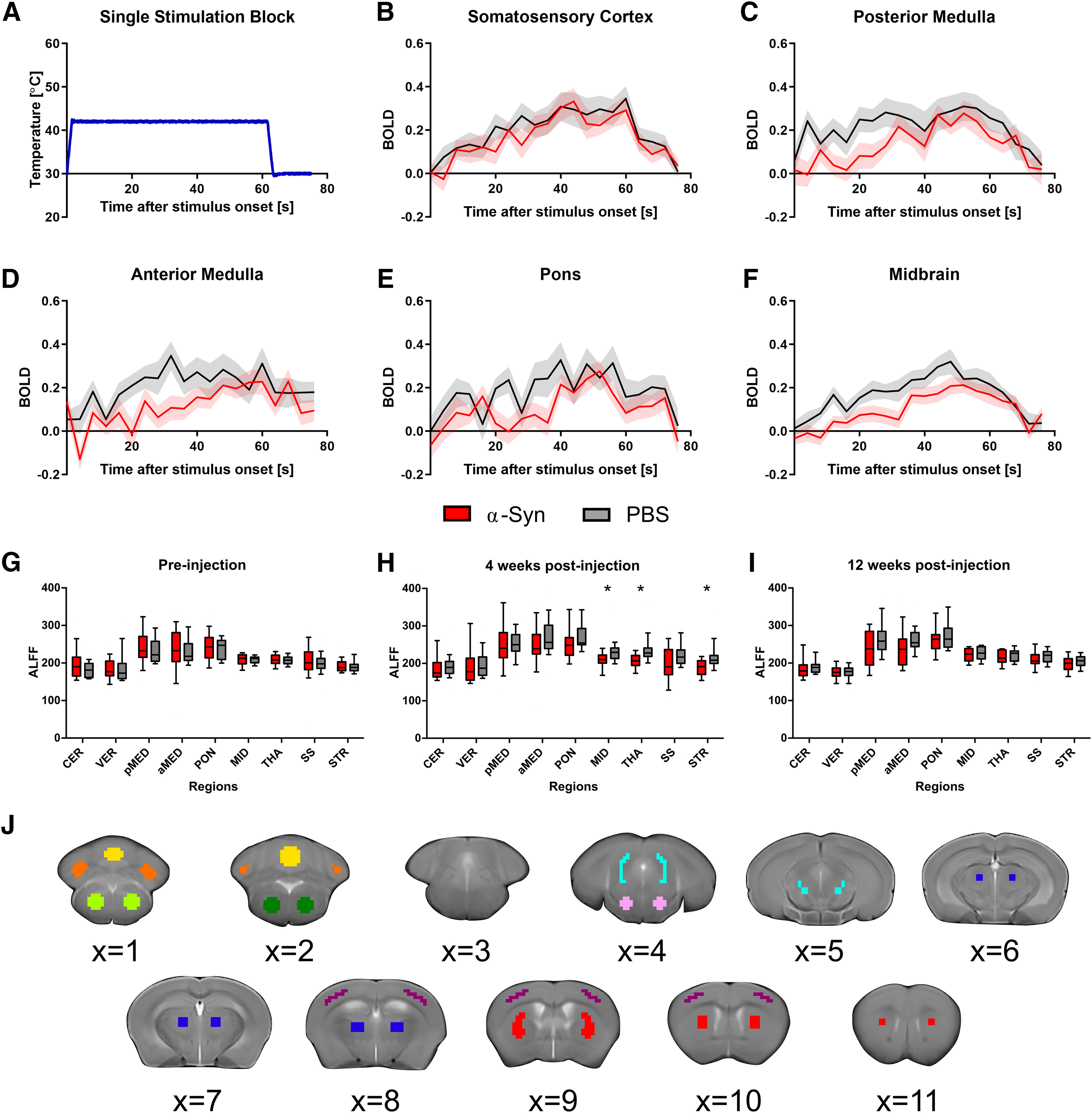
Intramuscular injection of α-syn fibrils induces reduced functional activity compared with PBS controls. ***A***, The temperature profile of one 60-s stimulation block applied to the plantar hind paw during the sensory-evoked fMRI scans. ***B***, BOLD signal in the somatosensory cortex as a result of the thermal stimulation profile given in ***A***. Signal was calculated at 4-s intervals from stimulation onset until 16 s after simulation termination. The somatosensory cortex confirms a robust increase in the BOLD signal followed by a decrease in the BOLD signal at stimulation termination. Semi-transparent region designates SEM. ***C–F***, At 12 weeks post-injection, mice that were inoculated with α-syn fibrils had significantly reduced BOLD signal in the (***C***) posterior medulla, (***D***) anterior medulla, (***E***) pons, and (***F***) midbrain compared with PBS controls. Semi-transparent region designates SEM. ***G–I***, Box plots show the interquartile range for the ALFF measured using resting-state fMRI (***G***) pre-injection, (***H***) four weeks post-injection, and (***I***) 12 weeks post-injection. ALFF was calculated in α-syn and PBS control mice in ROIs: cerebellum (CER), vermis (VER), posterior medulla (pMED), anterior medulla (aMED), pons (PON), midbrain (MID), thalamus (THA), somatosensory cortex (SS), striatum (STR). Regions with significant differences between groups after FDR correction are designated with asterisk(s) (*p_fdr_ < 0.05; **p_fdr_ < 0.01). ***J***, ROIs used in fMRI analyses were derived from the dMRI ROIs by linear registration of the B0 template to the T2 anatomic template. Regions were drawn on and are displayed overlaid over the T2 anatomic template: cerebellum (orange), vermis (yellow), posterior medulla (light green), anterior medulla (dark green), pons (pink), midbrain (turquoise), thalamus (dark blue), somatosensory cortex (purple), striatum (red).

A T2 anatomic template was created for the registration of fMRI images. To create the template image, 160 T2 anatomic images were iteratively averaged, registered to the average, then averaged again using the antsMultivariateTemplateConstruction.sh script in the ANTs toolkit. This custom template allowed us to register our images to a common coordinate space while minimizing the magnitude of the transformations calculated, thus maximizing the registration accuracy. ROIs used in the fMRI analyses were derived from the dMRI template ROIs so that the same regions examined in the dMRI analysis spatially matched those examined in the fMRI analysis. To create the T2 anatomic template ROIs, the dMRI template was linearly registered to the T2 anatomic template and the calculated transformations were applied to the dMRI template ROIs to warp these ROIs into T2 anatomic space. The ROIs were then resampled to match the resolution of the fMRI scans using nearest-neighbor interpolation, and a threshold of 0.5 was applied to binarize the ROIs. The resultant T2 anatomic template ROIs are shown in Figure 2*B* overlaid on the representative temporal average of the resting-state fMRI scans and Figure 4*J* overlaid on the T2 anatomic template.

#### Sensory-evoked fMRI analysis

The stimulus-related hemodynamic response time series was modeled using the hemodynamic response function convolved with 19 “tent” or piecewise linear B-spline basis functions (Saad et al., 2006) spanning 76 s from the onset of each stimulation block. Preprocessed images were regressed to these simulated response functions. Six motion parameters calculated during the motion correction step (three rotational and three translational) were included in the general linear model as regressors of no interest. Volumes flagged to be censored by 3dToutcount or for excessive motion were censored in this step by exclusion from the regression analysis. The dependent variables at this level of the analysis were the estimated β-coefficients (one for each tent regressor) of the regressed time series and their associated *t* statistics.

The temporal mean of the functional scan was nonlinearly registered to T2 anatomic subject space and then to T2 anatomic template space. The calculated transformations were applied to the β-coefficient and *t* statistic maps. ROIs in T2 anatomic template space were applied to the β-coefficient maps; however, the striatum ROI was excluded from the sensory-evoked fMRI analysis because the striatum was not found to have between-group microstructural differences at any of the timepoints according to the dMRI analysis. Additionally, the striatum has been shown to be affected by neither α-syn pathology nor inflammation in this α-syn mouse model (Sacino et al., 2014; Sorrentino et al., 2018). Bilateral ROIs were applied to capture activity from unilateral stimulation because unilateral heat stimulation has been previously shown to result in bilateral fMRI activity in mice (Bosshard et al., 2015). Mean BOLD values were then calculated within each ROI to create a BOLD response time series for each ROI for each scan.

#### Resting-state fMRI analysis

A regression analysis was used to remove the effects of motion from the resting-state signal. Twelve motion parameters (three translational, three rotational, and their first order temporal derivatives) calculated during the motion correction step were included in the general linear model as regressors (Satterthwaite et al., 2013). The residual time series contains the filtered signal with the effects of motion removed. The amplitude of low-frequency fluctuations (ALFF) and fractional ALFF maps (0.01–0.1 Hz) were then calculated from the residual time series using the 3dRSFC program from the AFNI toolbox (Zang et al., 2007; Zou et al., 2008; Taylor and Saad, 2013). ALFF calculation requires a constant time series so TRs were not censored in the preceding regression step. The temporal mean of the functional scan was nonlinearly registered to T2 anatomic subject space and then to T2 anatomic template space. The calculated transformations were applied to the ALFF and fractional ALFF maps. Group average ALFF maps in T2 anatomic template space are shown in Figure 2*C* and illustrate the image quality and registration quality. ROIs in T2 anatomic template space were applied and the mean ALFF and fractional ALFF values were then calculated within each ROI.

#### Statistical analysis

Statistical analyses were performed with R version 3.3.2 (http://www.R-project.org) and SPSS Statistics 25 (IBM). Between-group effects (α-syn injected vs PBS injected) were calculated for dMRI (fractional anisotropy and mean diffusivity) and resting-state fMRI (ALFF and fractional ALFF) metrics using ANCOVAs covaried for sex. Between-group effects (α-syn injected vs PBS injected) in rotarod (latency to fall) and sensory-evoked fMRI (BOLD time series) were analyzed using repeated-measures ANCOVAs with sex included as a covariate. All results were corrected for multiple comparisons using the false discovery rate (FDR) method at *p* < 0.05 (Benjamini and Hochberg, 1995). To interpret effect size, Cohen's *d* was calculated for significant FA and ALFF between-group differences (Sullivan and Feinn, 2012).

To determine which metrics and which regions within each metric best predicted survival time, four Cox proportional hazards regression models (Cox, 1972) were created on the metrics in which significant group differences were found (fractional anisotropy at 12 weeks post-injection, sensory-evoked BOLD at 12 weeks post-injection, fractional anisotropy at four week post-injection, and ALFF at four weeks post-injection). All mice that did not display symptoms of hindlimb paralysis were censored in the analysis as surviving beyond 170 d post-injection. Sex was included as a covariate in the regression models. Data were quartile split to reduce the effects of overfitting.

## Results

### Intramuscular injection of α-syn fibrils in M83^+/−^ mice results in bilateral paralysis and death

M83^+/−^ mice intramuscularly injected with preformed α-syn fibrils display reduced survival time because of the presence of severe hind leg paralysis (Ayers et al., 2017; Sorrentino et al., 2018) that requires euthanasia (117 ± 6 d post-injection) compared with PBS control mice. PBS control mice were monitored for 170 d post-injection and were all confirmed to survive beyond this age (Fig. 3*A*). A rotarod assessment was performed to quantify the progression of locomotor deficits however no significant between-group differences in latency to fall were found at any of the three timepoints assessed (Fig. 3*B*).

### Intramuscular injection of α-syn fibrils in M83^+/−^ mice induces microstructural changes at four and 12 weeks post-injection

dMRI is an *in vivo* imaging technique for measuring microstructural differences between M83^+/−^ mice intramuscularly inoculated with α-syn fibrils and PBS control mice. No differences in mean diffusivity were found at any of the timepoints between the mice injected with α-syn fibrils and PBS control mice. Pre-injection, it was confirmed that no between-group differences in fractional anisotropy were found (Fig. 3*C*). Four weeks post-injection, the mice injected with α-syn fibrils had reduced fractional anisotropy (*p* < 0.05; FDR corrected) in the cerebellum, vermis, anterior medulla, posterior medulla, and somatosensory cortex compared with PBS control mice (Fig. 3*D*).The Cohen's *d* for these effects were calculated to be 1.74, 1.87, 1.03, 1.18, and 1.90, which correspond to very large, very large, large, large, and very large effect sizes, respectively (Sullivan and Feinn, 2012). Twelve weeks post-injection, the mice injected with α-syn fibrils had reduced fractional anisotropy (*p* < 0.01; FDR corrected) in the pons and the thalamus compared with PBS control mice (Fig. 3*E*).The Cohen's *d* for these effects were calculated to be 1.20 and 1.09 which both correspond to large effect sizes (Sullivan and Feinn, 2012).

### Intramuscular injection of α-syn fibrils in M83^+/−^ mice induces reduced sensory activation at 12 weeks post-injection

fMRI during 60-s blocks of thermal heat stimulation (Fig. 4*A*) of the hindlimb was collected on mice injected with α-syn fibrils and PBS control mice at all three timepoints, and the temporal progression of the BOLD signal was quantified for 76 s after stimulation onset. It was confirmed that a robust increase of the BOLD signal was observed in the somatosensory cortex as a result of 60 s of thermal stimulation, followed by a reduction in the BOLD signal at termination of the sensory stimulation (Fig. 4*B*). Additionally, it was confirmed that pre-injection, there were no significant between-group differences in the BOLD signal. Four weeks post-injection, no significant between-group differences in BOLD signal were found. Twelve weeks post-injection, mice injected with α-syn fibrils had a reduced BOLD response in the posterior medulla, anterior medulla, pons, and midbrain compared with PBS-injected controls (*p* < 0.05; FDR corrected). The average BOLD signal when the signal peaks in the posterior medulla, anterior medulla, pons, and midbrain for the mice injected with α-syn fibrils at 12 weeks post-injection was 0.278, 0.229, 0.276, and 0.212, respectively. For PBS controls at 12 weeks post-injection, the average BOLD signal when the signal peaks in the posterior medulla, anterior medulla, pons, and midbrain was 0.310, 0.347, 0.327, and 0.321, respectively; *p* values for the effect of group on the BOLD signal in the examined regions at each time point are given in Table 1. Figure 4*C–F* depicts the calculated BOLD time series for regions in which significant group differences were found.

**Table 1.** Thermal fMRI BOLD between-group effects

ROI	Pre-injection	4 weeks post-injection	12 weeks post-injection
Cerebellum	0.513	0.525	0.334
Vermis	0.303	0.525	0.428
Posterior medulla	0.154	0.776	0.048*
Anterior medulla	0.148	0.525	0.048*
Pons	0.148	0.776	0.032*
Midbrain	0.148	0.525	0.048*
Thalamus	0.148	0.525	0.095
Somatosensory cortex	0.943	0.862	0.091

A repeated-measures ANCOVA was performed on the 20 BOLD signal values for each ROI. Sex was included in the statistical models as a covariate. The table presents the FDR corrected p-values for between-group effects.

### Intramuscular injection of α-syn fibrils in M83^+/−^ mice induces reduced spontaneous activity at four weeks post-injection

Resting-state fMRI scans were collected for both mice injected with α-syn fibrils or PBS at all three timepoints. ALFF and fractional ALFF were calculated to quantify the spontaneous activity of regions throughout the brain while at rest. No differences in fractional ALFF were found between groups at any of the three timepoints. It was confirmed that pre-injection, no group differences in ALFF were found (Fig. 4*G*). Four weeks post-injection of α-syn fibrils, mice had reduced ALFF compared with PBS controls in the midbrain, thalamus, and striatum (*p* < 0.05; FDR corrected; Fig. 4*H*). The Cohen's *d* for these effects were calculated to be 1.10, 1.36, and 1.24, which correspond to large, very large, and large effect sizes, respectively (Sullivan and Feinn, 2012). No between-group differences were found in the examined regions at 12 weeks post-injection (Fig. 4*I*).

### Fractional anisotropy in the pons is predictive of survival time

To examine which MRI metrics and which regions in each MRI metric were predictive of survival time in the α-syn group, four Cox proportional hazards regression models were created. Each model included MRI metrics in which significant between-group differences were found: (1) fractional anisotropy at 12 weeks post-injection, (2) sensory-evoked BOLD at 12 weeks post-injection, (3) fractional anisotropy at four weeks post-injection, and (4) ALFF at four weeks post-injection. Out of the four corresponding regression models, only the fractional anisotropy at 12 weeks post-injection model had a statistically significant overall fit (*p* = 0.001). Within the fractional anisotropy at 12 weeks post-injection model, a reduction in fractional anisotropy in the pons was significantly associated with an increased risk of death (hazard ratio = 0.444; *p* < 0.05). Hazard ratios, 95% confidence intervals, and the quartile split thresholds for each region for each MRI metric are included in Table 2.

**Table 2.** Proportional hazards models

	Hazard ratio (95% CI)	Quartile thresholds		
		1st	2nd	3rd
12 Weeks post-injection; fractional anisotropy (*p* = 0.001)*				
Pons	0.444 (0.238–0.831)*	0.176	0.198	0.215
Thalamus	0.722 (0.419–1.244)	0.154	0.170	0.189
12 Weeks post-injection; BOLD (*p* = 0.289)				
Posterior medulla	0.920 (0.465–1.818)	0.097	0.160	0.223
Anterior medulla	0.756 (0.450–1.272)	0.067	0.139	0.230
Pons	0.690 (0.423–1.124)	0.077	0.149	0.183
Midbrain	1.030 (0.538–1.971)	0.078	0.123	0.206
4 Weeks post-injection; fractional anisotropy (*p* = 0.415)				
Cerebellum	1.059 (0.453–2.474)	0.141	0.153	0.165
Vermis	0.699 (0.326–1.502)	0.160	0.172	0.187
Posterior medulla	1.164 (0.625–2.168)	0.147	0.160	0.180
Anterior medulla	0.802 (0.383–1.681)	0.143	0.157	0.172
Somatosensory cortex	0.781 (0.428–1.426)	0.130	0.136	0.144
4 Weeks post-injection; ALFF (*p* = 0.100)				
Midbrain	0.559 (0.233–1.338)	207.733	214.685	229.870
Thalamus	1.059 (0.361–3.111)	201.753	218.889	228.060
Striatum	0.877 (0.413–1.863)	183.379	204.803	210.260

Four Cox proportional hazards models were created to determine the variables most correlated with survival time: 12 weeks post-injection dMRI fractional anisotropy, 12 weeks post-injection sensory-evoked fMRI BOLD signal, four weeks post-injection dMRI fractional anisotropy, and four weeks post-injection resting-state fMRI ALFF; *p*-values given in parentheses give the overall model significance. Only regions previously determined to have significant between-group differences were used in the models. Data were quartile split to reduce the effects of overfitting, and the quartile thresholds are given. The hazard ratio for each region is given along with the 95% confidence interval;

*p < 0.05.

## Discussion

The current study characterizes the progression of structural, functional activity, and behavioral changes induced by α-syn pathology. The major findings provide evidence for a transient microstructure and functional activity change in the brain resulting from the injection of α-syn fibrils four weeks prior. At 12 weeks post-injection, a pattern of microstructure and functional activity deficits was observed that overlaps with previously reported locations (pons and thalamus) of α-syn pathology and immune activation (Sorrentino et al., 2018). Additionally, these changes detected by *in vivo* imaging preceded measurable locomotor deficits and reduced fractional anisotropy in the pons was determined to be predictive of shorter survival time. Altogether, this body of work suggests that α-syn inclusion pathology can have systemic effects on the function and structure of the mouse brain, and that unique patterns of functional and structural changes occur at different stages of α-syn pathology progression.

Diffusion tensor metrics have been shown to be sensitive to microstructural changes in gray matter regions (Lythgoe et al., 1997; Hoehn-Berlage et al., 1999; Péran et al., 2010; Pfefferbaum et al., 2010; Du et al., 2011) and white matter tracts (Le Bihan et al., 2001; Madden et al., 2009; Ji et al., 2015; Chiang et al., 2017). Diffusion measures have been shown to correlate with 1-methyl-4-phenyl-1,2,3,6-tetrahydropyridine (MTPT) dose, nigral tyrosine hydroxylase (TH)-positive cell bodies, and striatal TH-positive fiber density in an MPTP macaque model (Shimony et al., 2018). Reduced fractional anisotropy has been associated with demyelination, neurodegeneration, dendritic disorganization, and edema (Le Bihan et al., 2001; Alexander et al., 2007). Our observation of reduced fractional anisotropy in mice inoculated with α-syn seeds compared with controls at four and 12 weeks post-injection (Fig. 3*D*,*E*) aligns with previous dMRI studies in humans with synucleinopathies that have reported reduced fractional anisotropy in Parkinson's disease (Vaillancourt et al., 2009; Péran et al., 2010; Du et al., 2011), Lewy body dementia (Kantarci et al., 2010; Watson et al., 2012), and multiple system atrophy (Shiga et al., 2005; Ito et al., 2007; Wang et al., 2011a) patients compared with healthy controls. Reduced fractional anisotropy has also been reported in MPTP (Boska et al., 2007) and lesion (Van Camp et al., 2009) Parkinson's disease murine models. It is important to note that fractional anisotropy was not different between groups before the injection of the α-syn fibrils.

Reduced fractional anisotropy was observed in the pons and thalamus at 12 weeks post-injection. These regions align with a previous histologic analysis of the same α-syn transmission mouse model that reported α-syn pathology in the pons and thalamus among other regions at 12 weeks post-injection (Sorrentino et al., 2018). Astrocytosis and microgliosis were also detected in the pons at 12 weeks post-injection (Sorrentino et al., 2018). At four weeks post-injection, reduced fractional anisotropy was observed in the cerebellum, vermis, posterior medulla, anterior medulla, and somatosensory cortex. Interestingly, reduced fractional anisotropy was not observed in these regions at 12 weeks post-injection of α-syn seeds compared with PBS controls. The recovery of fractional anisotropy in the hindbrain and cortex at 12 weeks post-injection suggests that reduced fractional anisotropy in the hindbrain and cortex at four weeks post-injection is not a result of neurodegeneration but may instead be an acute inflammation effect. Inflammation has been shown to play an important role in the etiology of Parkinson's disease (McGeer and McGeer, 2008). Additionally, reduced fractional anisotropy has been found to correlate with activated microglia in traumatic brain injury patients (Scott et al., 2015), reduced T-cell count in chronic HIV infection patients (Wright et al., 2015), leukocyte apoptosis in sleep apnea patients (Chen et al., 2015), leukocyte adhesion molecules in Parkinson's disease (PD) patients (Chiang et al., 2017), and leukocyte apoptosis in PD patients (Chiang et al., 2017). Together, these results reveal distinct patterns of structural changes at four and 12 weeks after intramuscular injection of pathologic α-syn that primarily progress anteriorly and precedes measurable locomotor deficits.

ALFF has been used to describe the intensity of spontaneous brain activity (Zang et al., 2007). Our observation of reduced ALFF in the striatum of α-syn seeded mice compared with PBS controls (Fig. 4*H*) aligns with previous resting-state fMRI studies in humans that have reported reduced ALFF in the putamen in PD patients compared with healthy controls (Wen et al., 2013; Tang et al., 2017) and a negative correlation between ALFF in the putamen and disease severity (Skidmore et al., 2013). Interestingly, ALFF group differences were not significant at 12 weeks post-injection (Fig. 4*I*). The transiently reduced ALFF mirrors the transient reduction in fractional anisotropy in the hindbrain and somatosensory cortex of α-syn seeded mice compared with PBS controls. These temporally coupled structural and functional abnormalities occurred as a result of the intramuscular α-syn injection, however, further work is needed to elicit the mechanism for the recovery of these metrics at 12 weeks post-injection.

fMRI during thermal stimulation is a useful assay for quantifying neuronal activity abnormalities in sensory processing. Our results indicate reduced activation in the hindbrain in α-syn seeded mice compared with PBS controls at 12 weeks post-injection (Fig. 4*C–F*). These results align with a thermal stimulation fMRI study that reported reduced brain activation in early drug-naive Parkinson's disease patients compared with controls (Tan et al., 2015). Reduced thermal stimulation BOLD signal in α-syn seeded mice compared with PBS controls occurred in the medulla, pons, and midbrain and concurrently with reduced fractional anisotropy in the pons and thalamus. It has also been reported that 12 weeks post-injection α-syn pathology was observed in the pons, midbrain, thalamus, and cortex while astrocytosis and microgliosis were observed in the pons (Sorrentino et al., 2018). Together, these results and prior literature reveal structural and functional abnormalities that are co-localized with α-syn pathology and, to a lesser extent, with immune activation.

To determine whether specific changes in functional activity and microstructure predict survival time, Cox proportional hazards models were created (Table 2). Fractional anisotropy in the pons at 12 weeks post-injection was the only metric found to be significantly predictive of survival time. At 12 weeks post-injection, the pons is also the only region in which structural group differences, functional group differences, α-syn pathology, and immune activation were all observed (Sorrentino et al., 2018). Together, this body of evidence suggests that the pons is consistently affected in this α-syn fibrils transmission mouse model at different levels of analysis. According to the Braak model (Braak et al., 2004), the pons is one of the first brain regions affected in Parkinson's disease and REM behavior sleep disorder (Boeve et al., 2007). The pons is also a hallmark region affected in the cerebellar variant of multiple system atrophy (Jellinger et al., 2005). An interesting observation here and in prior work (Sorrentino et al., 2018) is that α-syn pathology and neurodegeneration do not always coincide, and this is consistent with predictions by prior work (Surmeier et al., 2017). Additionally, these results show that between dMRI, resting-state fMRI, and sensory-evoked fMRI, dMRI is the only method that associated with survival time. These patterns of change also occurred despite no measurable behavioral deficits in the mice at 12 weeks of age, suggesting that the *in vivo* imaging changes occurred before the behavioral phenotype.

There are a few limitations that must be considered when interpreting the results of this study. Although many fMRI studies have examined the brainstem and cerebellum (Komisaruk et al., 2002; Bense et al., 2006; Fougère et al., 2010), the fMRI signal in these areas is reduced by the presence of white matter and could be influenced by physiological noise. To reduce the effects of physiological noise, six motion parameters were added as nuisance regressors in the deconvolution portion of the fMRI analysis. It is unknown whether the observed between-group differences are unique to α-syn pathology compared with other proteinopathies. Reduced FA has been reported in Aβ (Colon-Perez et al., 2019), tau (Colgan et al., 2016), and SOD1 (Marcuzzo et al., 2017) mouse models compared with controls. Also of note, areas of increased and reduced ALFF have been found in mild cognitive impairment and Alzheimer's disease patients (Wang et al., 2011b; Dai et al., 2012).

In this study, we characterized the progression of structural and functional changes occurring in the brain as a result of α-syn pathology progression. The major findings from this study suggest that MRI-detectable structural and functional changes in the brain of α-syn seeded mice occur as early as four weeks post-injection. At 12 weeks post-injection, a separate and distinct pattern of structural and functional changes were observed that are co-localized with previously reported regions of α-syn pathology and, to a lesser extent, immune activation. Finally, fractional anisotropy in the pons was identified as a predictor of survival time. The major findings from this study provide evidence that diffusion and fMRI are useful *in vivo* markers of α-syn pathology disease progression with potential applications in preclinical drug screening. Additionally, these findings provide preclinical evidence in support of the use of diffusion and fMRI as markers relevant to synucleinopathy in humans.
